# Waveguide-Coupled Light Photodetector Based on Two-Dimensional Molybdenum Disulfide

**DOI:** 10.1021/acsami.4c04854

**Published:** 2024-05-25

**Authors:** Daria Hlushchenko, Jacek Olszewski, Tadeusz Martynkien, Michał Łukomski, Karolina Gemza, Pawel Karasiński, Magdalena Zięba, Tomasz Baraniecki, Łukasz Duda, Alicja Bachmatiuk, Małgorzata Guzik, Robert Kudrawiec

**Affiliations:** † Łukasiewicz Research Network-PORT Polish Center for Technology Development, ul. Stabłowicka 147, 54-066 Wrocław, Poland; ‡ Faculty of Fundamental Problems of Science and Technology, Wrocław University of Science and Technology, Wybrzeże Wyspiańskiego 27, 50-370 Wrocław, Poland; § Department of Optoelectronics, Silesian University of Technology, ul. B. Krzywoustego 2, 44-100 Gliwice, Poland; ∥ Faculty of Chemistry, University of Wrocław, ul. F. Joliot-Curie 14, 50-383 Wrocław, Poland

**Keywords:** MoS_2_, doped two-dimensional materials, photodetector, mechanical exfoliation, transition metal dichalcogenides, optical waveguide, sol–gel

## Abstract

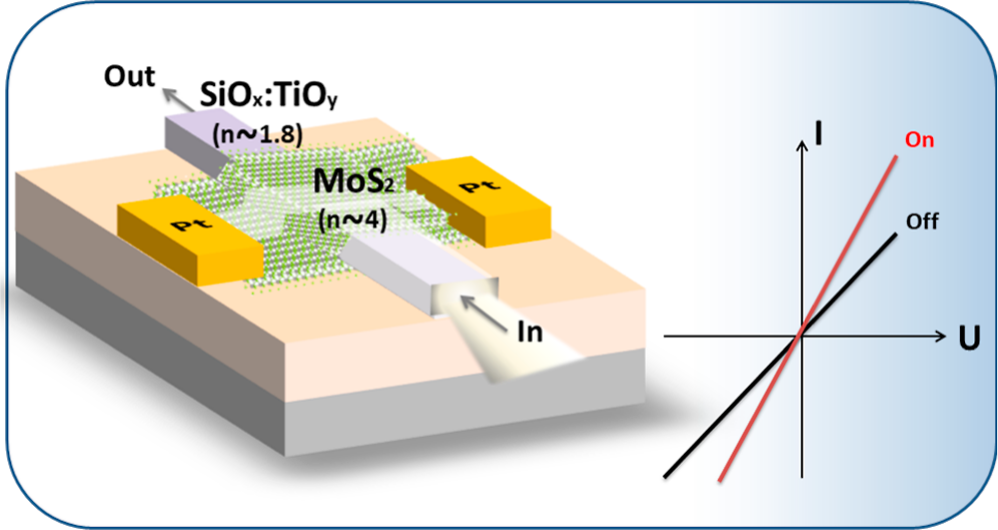

The integration of transition metal dichalcogenides with photonic structures such as sol–gel SiO_*x*_:TiO_*y*_ optical waveguides (WGs) makes possible the fabrication of photonic devices with the desired characteristics in the visible spectral range. In this study, we propose and experimentally demonstrate a MoS_2_-based photodetector integrated with a sol–gel SiO_*x*_:TiO_*y*_ WG. Based on the spectroscopic measurements performed for our device, we concluded that the light entering the WG is almost completely channeled out from the WG and absorbed by the MoS_2_ flake, which is deposited on the WG. Therefore, this device works as a photodetector. The light coupling into the MoS_2_ region in this device construction is due to the high contrast of refractive index between the van der Waals crystal and the sol–gel WG, which is ∼4 and ∼1.8, respectively. The obtained MoS_2_-based photodetectors exhibit a photoresponsivity of 0.3 A W^−1^ (*n*-type MoS_2_) and 7.53 mA W^−1^ (*p*-type MoS_2_) at a bias voltage of 2 V. These results reveal great potential in the integration of sol–gel WGs with van der Waals crystals in optoelectronic applications.

## 1 Introduction

Strong application possibilities characterize the transition metal dichalcogenide (TMD)-based photonic devices.^1−3^ The properties of TMDs permit for the fabrication of waveguide (WG)-integrated devices such as photodetectors,^4−22^ modulators,^23^ quantum emitters,^24−26^ and integrated circuits.^27−30^ The combination of WGs and optical modulators, which is already used for electrical-to-optical conversions, is a manner to find alternatives to commercially produced copper interconnectors in complementary metal–oxide–semiconductor (CMOS) transistors.^28,29^ The major disadvantage of these types of transistors is the heating of on-chip copper interconnectors.^10,11,17^ Therefore, the fabrication of optical TMD devices on top of WGs can be an alternative solution to this problem.

TMD materials are characterized by extensive optical parameters, suitable carrier mobility, and appropriate absorption coefficient.^1,6,31,32^ The composition and number of layers can be used to control the optical response. Excellent electrically tunable physical properties are ideal for creating functionalized photoelectric information devices.^33−39^ The most explored materials for these types of applications are molybdenum disulfide (MoS_2_),^33−42^ platinum disulfide (PtS_2_),^35−37,43−46^ molybdenum ditelluride (MoTe_2_),^35,47^ and mixed heterostructures.^48−51^

The basic principle of photodetectors and other optoelectronic devices relies on converting the absorbed photons into electrical signals.^18^ During this conversion, a few processes occur like light harvesting, exciton dissociation, and transport of charge carrier to metal electrodes.^18^ Various mechanisms, such as the photovoltaic effect, photoconductive effect, photothermoelectric effect, bolometric effect, and surface plasmonic effect have been reported for photodetection mechanisms in photodetectors and optoelectronic devices.^2,4,7,9,15,17,35,39^

During measuring the device characteristics, it is also important to take into account the effect of persistent photoconductivity, where the device remains in an excited state for a longer time duration even in dark conditions.^52−56^ Under the flake illumination, the photoinduced desorption of O_2_ and H_2_O molecules from the flake surface appears, which is also observed in MoS_2_ devices.^53−56^ In this work, the device was checked for the appearance of a persistent photoconductivity.

Sol–gel SiO_*x*_:TiO_*y*_ layers are a novel platform for developing photonic structures such as WGs, ring resonators, or integrated circuits.^57^ Sol–gel SiO_*x*_:TiO_*y*_ layers (refractive index *n* ∼ 1.8) have been used as a WG platform owing to their transparency for frequencies in the visible spectral range, good transmission properties, and low optical losses.^57^ Because of these advantages, a sol–gel material platform was used as the basis for optical WG fabrication. But so far, the use of sol–gel technology to produce WGs coupled with detectors has not been reported. Moreover, there are no reports of van der Waals crystal-based devices containing elements (protective or reflective layers, WGs, or other passive elements) made of SiO_*x*_:TiO_*y*_ in the sol–gel technology. In this context, the combination of these two material systems in optoelectronic devices, including detectors, is very intriguing. This is even more interesting because these two material systems (*i.e.*, SiO_*x*_:TiO_*y*_ glasses and van der Waals crystals) can differ very significantly in their refractive index.

In this study, we demonstrate a proof-of-principle device in which light propagates along a SiO_*x*_:TiO_*y*_ sol–gel WG and then is converted to an electrical signal using a MoS_2_-based photodetector, which is integrated with this WG. To understand the operation of such a device, prototypes with different absorber thicknesses were made, their current–voltage characteristics were measured, and measurements of transmission through WGs, as well as the simulations of light propagation, were performed. This allowed us to understand the operation of this kind of device and evaluate the prospects for developing these types of photodetectors. This new detector concept is based on the high contrast of the refractive index between the WG and the active part, opens the possibility of further optimization of these types of detectors, and can be used to construct detectors with a similar concept in other material systems.

## 2 Results and Discussion

### 2.1 Fabricated Devices on the Sol–Gel SiO_*x*_:TiO_*y*_ WG

**Figure 1:**
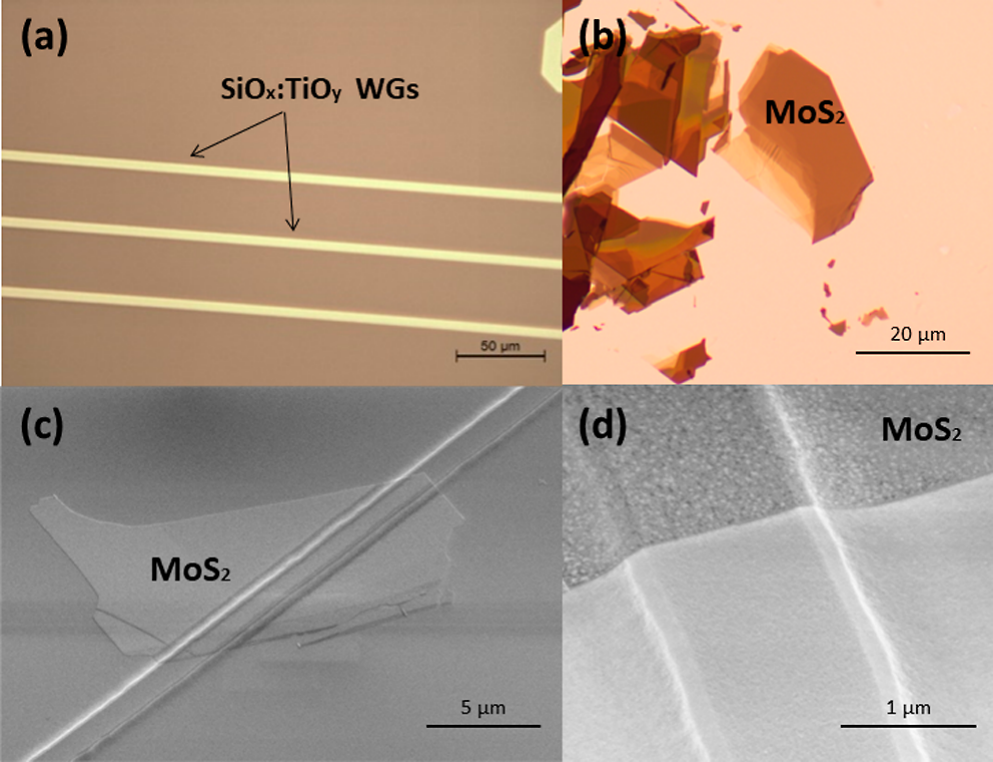
(a) Optical image of the fabricated sol–gel SiO_*x*_:TiO_*y*_ WGs. The scale bar is 50 μm. (b) View of exfoliated MoS_2_ flakes in an optical microscope in reflected mode. (c) Scanning electronic microscopy (SEM) image of the transferred MoS_2_ flake on top of the WG. (d) Zoomed view of the MoS_2_ few layer on top of the WG.

The fabricated samples consisted of several sol–gel SiO_*x*_:TiO_*y*_ WGs (Figure 1a). The width and height of the WGs were 1.2–5 and 0.270 μm, respectively. Based on the optical contrast between MoS_2_ and substrate, flakes were selected and transferred onto the top of sol–gel WGs (Figure 1c,d).

**Figure 2:**
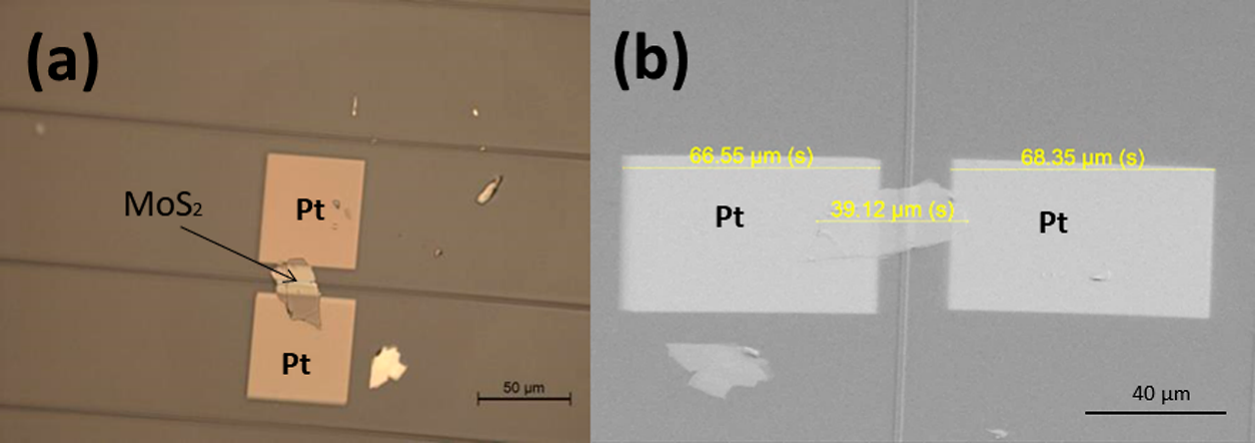
(a) Image of fabricated Pt contacts (viewed from an optical microscope). (b) Zoom-in view of the device with contacts from SEM.

Gas injection system (GIS) deposition with an organometallic platinum (CH_3_)_3_Pt(C_p_CH_3_) precursor was used to fabricate the platinum (Pt) pads (Figure 2). The platinum precursor is decomposed using a focused ion beam (FIB) during contacts deposition. The FIB in this method aids in removing the oxide layers on the TMD surface and prevents the formation of Schottky contact.^58^ These types of metal pads were used for electrical probing, providing good charge conduction and low contact resistance for the device.^58^ In all photodetectors tested in this work, Pt contacts with dimensions of 65 × 50 μm^2^ and a thickness of approximately 50 nm were deposited, and Ohmic contacts were obtained in all cases. Obtaining Ohmic contacts is the main reason for using this contact preparation technology and Pt as a material with high work function. Additionally, Pt precursor for contact deposition is widely used in FIB, and such contacts can be made intentionally in a given place without lithographic masks, which is a great advantage in the case of van der Waals crystal flakes with irregular shapes. In general, other Ohmic contact technologies can also be used in this case.

### 2.2 Principle of Photodetector Operation

Several photodetectors, created on van der Waals crystals, operate on the photoconductive effect, where the semiconductor conductivity changes under the influence of incident light.^17−21,23,29^

**Figure 3:**
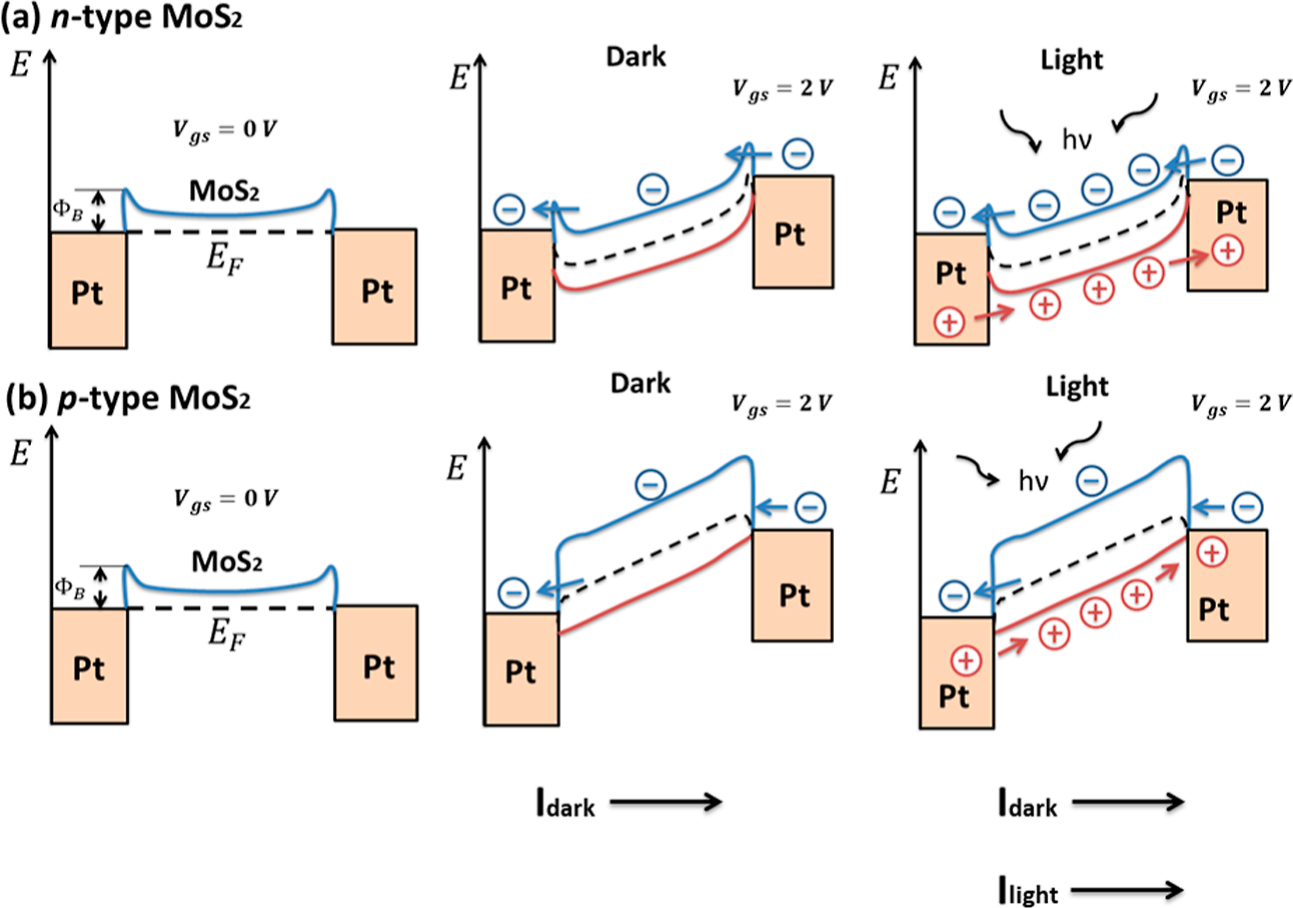
Schematic of the photoconductive effect in a photodetector based on (a) *n*-type MoS_2_ and (b) *p*-type MoS_2_. From left to right: energy diagram of a MoS_2_ semiconductor with two Pt contacts for unbiased condition; energy diagrams of the same device after applying a bias voltage in the dark (*I*_dark_) and after coupling light from the WG (*I*_light_).

When the contact is formed between the MoS_2_ material and metal, the band structures will change due to the alignment of the Fermi levels.^59−61^ The conduction and valence band edge of *p*-MoS_2_ will bend down (Figure 3b). On the contrary, the conduction and valence band edge of *n*-MoS_2_ will bend up (Figure 3a). Then, the energy barrier will be generated between the metal and semiconductor.

When a bias voltage (−2 V ≤ *V*_gs_ ≤ 2 V) was applied to the device in dark conditions, a dark current (*I*_dark_) appeared between the two metal electrodes (Figure 3). Further, the device is exposed to light from the WG with a photon energy higher than the bandgap value and begins to conduct extra current. The photogenerated electron–hole pairs are separated due to the applied bias, or they can form excitons, which have to be dissociated to contribute to the extra current. Finally, the drift of electrons and holes in different directions increased the current (*I*_light_) between the metal pads. The device conductance changes due to photoabsorption: the minority carriers are stacked in localized states, which change the Fermi level and induce majority carriers, as shown in Figure 3. Thus, the device can achieve a large photogain with a high photoresponse.^17,35^ Additionally, it is worth noting that the Joule heat generated in this detector at the maximum voltage is below a few milliwatts and does not significantly affect the properties of the flake (*i.e.*, temperature and absorption).

### 2.3 Electro-optical Photodetector Characterization

After the MoS_2_ flake deposition on the sol–gel WG, each device was probed with two-terminal transport measurements using platinum electrical contacts to analyze the photoresponsivity and dark current. A bias voltage was applied between the two inner contacts for the two-point measurement configuration, and the current flowing through them was measured.

**Figure 4:**
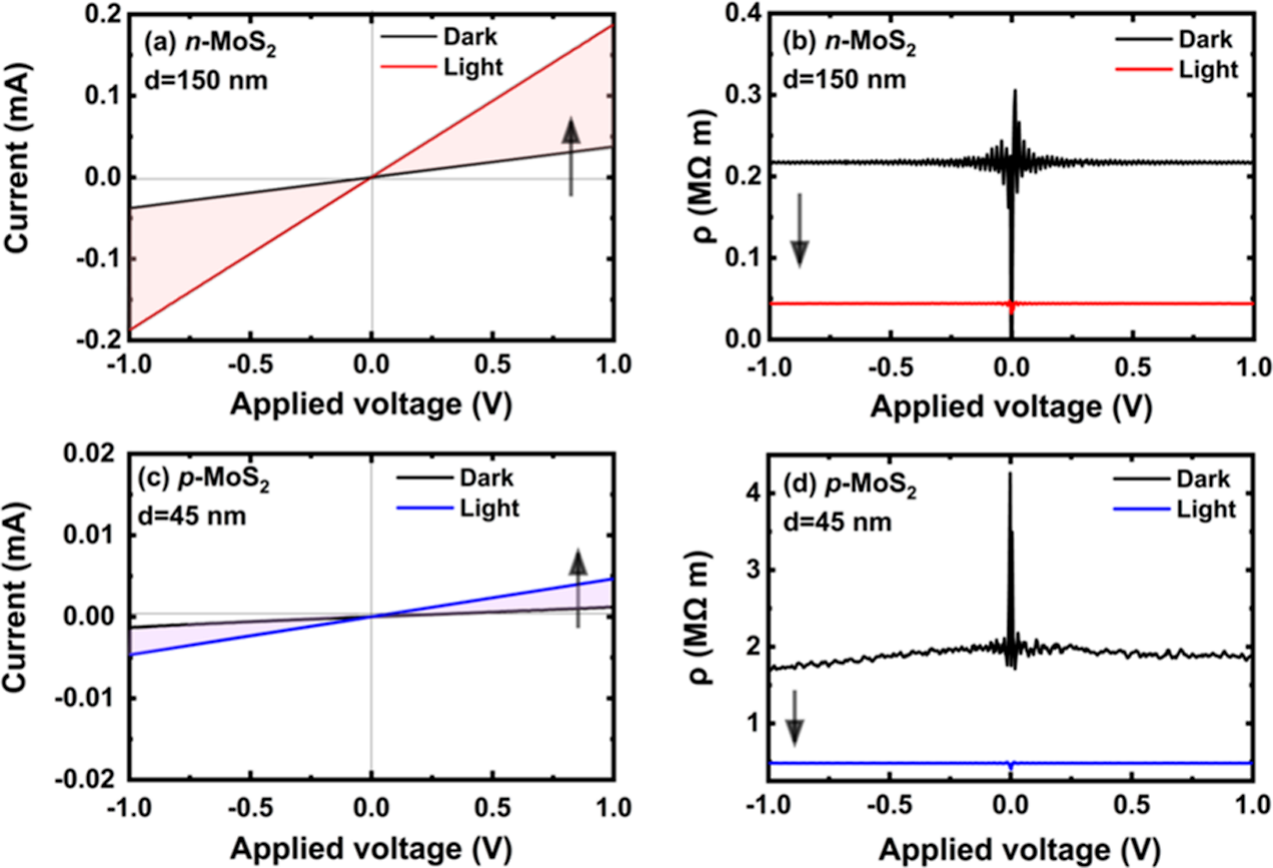
Optoelectrical photodetector characterization: (a) *I–V* characteristics for *n*-MoS_2_/WG (a flake thickness of *d* = 150 nm). (b) Calculated *ρ–V* characteristics for *n*-MoS_2_/WG. (c) *I*–*V* characteristics for *p*-MoS_2_/WG (flake thickness *d* = 45 nm). (d) Calculated *ρ–V* characteristics for *p*-MoS_2_/WG. A broad-band light source power of 100 μW was used for all measurements.

Figure 4 shows the room-temperature current–voltage (*I*–*V*) Ohmic characteristics of a representative photodetector with *n*- and *p*-type MoS_2_ active parts. In this case, the photodetector characterization is as follows: *I*–*V* curves were first measured without light-to-WG coupling (*I*_dark_) and then repeated with light-to-WG coupling (*I*_light_). As can be clearly seen in this figure, when the light is introduced into the WG, an additional current, *i.e.*, photocurrent, is generated in MoS_2_, and hence, higher current is observed in the *I*–*V* characteristics (see Figure 4a,c) and lower resistivity is determined from the *I*–*V* characteristics (see Figure 4b,d). It is worth emphasizing that the measured *I*–*V* characteristics increased linearly with the increase in bias voltage, indicating a zero barrier at the Pt/MoS_2_ junction.

Comparison of the *I*–*V* characteristics for the manufactured devices based on *n*- and *p*-type MoS_2_ indicates that the *n*-type MoS_2_-based device is characterized by higher photocurrent values in the *I*_light_ mode. When the concentrations of free carriers are very similar in *n*- and *p*-type MoS_2_, higher conductivity can be expected for the *n*-type material due to the higher electron mobility in MoS_2_^13,22,59^ but not by an order of magnitude. Therefore, the observed higher conductivity in *n*-type MoS_2_ is also related to the higher concentration of free carriers in this material.

Resistivity (*ρ*) is calculated as follows^34−36^
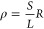
1where *R* denotes the resistance (calculated using *I*–*V* characteristics), *S* denotes the cross-sectional area of the exfoliated flake, which is perpendicular to the current flow, and *L* denotes the length of the MoS_2_ flake between the Pt pads. The cross-sectional area of the flake was calculated by formula: *S* = *wd*, where *w* and *d* denote the flake width and thickness, respectively.

**Figure 5:**
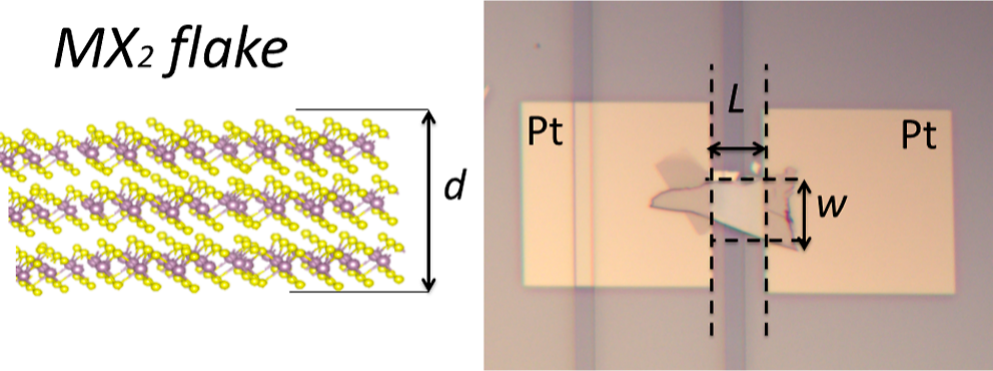
MX_2_ flake parameters (flake length between pads—*L*, width—*w*, and thickness—*d*).

Figure 5 represents the flake parameters (*L*, *w*, and *d*) for the calculation of the cross-sectional area.

According to the performance of the photodetectors, the main parameter is the photoresponsivity (*P*_R_), which describes the photoelectric conversion efficiency. The photoresponsivity is calculated as follows^15,36^
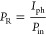
2where *I*_ph_ denotes the photocurrent and *P*_in_ is a power of the coupled light to the WG. The value defined in this way corresponds to external photoresponsivity because we are dealing with losses of light in the WG before reaching the van der Waals crystal flake, and additionally, not all light is absorbed by this flake. The generated photocurrent in the photodetector increases with the increasing of the coupled light to the WG and gate bias, and it is calculated using the following equation^4,7,12^
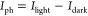
3

The values of photoresponsivity, which different groups have reported, are characterized widespread owing to the method of sample preparation (exfoliated TMDs or grown by CVD),^2,4,22,41^ type of TMDs,^4−7,15,21,34,39^ chemical doping,^17^ impurity and defect states, layer number,^17^ and type of substrate (Si, SiO_2_, SiC, GaAs, or other).^7,10,15^ In this study, we focused exclusively on the photoresponsivity of the device with the *n*- and *p*-type active material, noting that this parameter varies with both the size of the MoS_2_ flake and its properties, including conductivity without illumination. Table 1 presents a list of created photodetectors based on WGs. Most of the previously created photodetectors operate at a wavelength of 1550 nm.

**Table 1:** Performance Comparison of WG-Based Photodetectors Integrated with 2D Materials

2D material	material WG	bias (V)	spectral responsivity (A W^−1^)	wavelength (nm)	refs
h-BN/MoTe_2_	silicon	3	23 × 10^−3^	1310	(5)
MoSe_2_	metallic	1.5	18 × 10^−3^	742	(8)
PtSe_2_	silicon nitride	8	12 × 10^−3^	1550	(10)
PdSe_2_	silicon	6	20 × 10^−3^	1550	(19)
MoTe_2_	silicon	2	5 × 10^−3^	1160	(29)
PtSe_2_	silicon	5	1.3 × 10^−9^	1550	(46)
MoTe_2_	silicon	1	0.5	1550	(47)
*n*-MoS_2_	sol–gel SiO_*x*_:TiO_*y*_	2	0.3	532	this work
*p*-MoS_2_	sol–gel SiO_*x*_:TiO_*y*_	2	4 × 10^−3^	532	this work

In our devices, at *V*_gs_ = 2 V, the sensitivities reach a maximum value of ∼3 × 10^−1^ and ∼4 × 10^−3^ A W^−1^ for *n*-MoS_2_ and *p*-MoS_2_, respectively, when illuminated with a 532 nm light supercontinuum source with a power of 100 μW. This type of high photoresponsivity for *n*-MoS_2_ is comparable to the values obtained for photodetectors on Si,^11,13,18,19^ Ge,^12^ SiC,^13^ SiO_2_,^13^ Al_2_O_3_,^22^ and Si_3_N_4_^10,23,24^ platforms.

**Figure 6:**
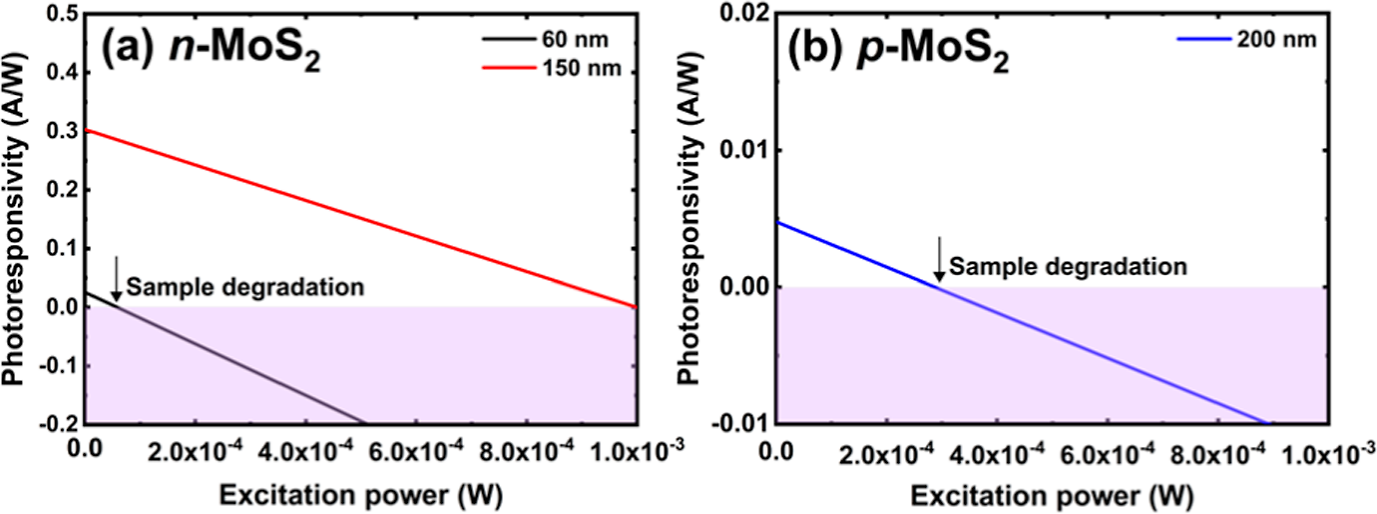
Dependence of photoresponsivity on the WG excitation power: (a) *n*-MoS_2_ (flake thickness *d* = 60 and 150 nm) and (b) *p*-MoS_2_ (flake thickness *d* = 200 nm). The results are presented for *V*_gs_ = 2 V. The WG was illuminated with a laser with a wavelength of 532 nm, increasing its power to 1 mW.

In general, detectors are expected to have a linear response with illumination power, which implies a constant photoresponsivity with power. However, in the case of detectors based on van der Waals crystals, changes in photoresponsivity with lighting power were observed.^3−7,10,13,17,21,22,36,37,51^ This phenomenon may be related to the degradation of van der Waals crystals under the influence of illumination and other phenomena typical of van der Waals crystals, which are worth investigating in further optimization of these types of detectors.^51^ To investigate this issue in our MoS_2_/WG detectors, light from a 532 nm laser was led into the WG, and its power was increased from 100 μW to 1 mW. Figure 6 shows the photoresponsivity as a function of the incident laser power at a bias of 2 V. The analysis of photoresponsivity shows a linear behavior for the photodetectors with *n*- and *p*-type MoS_2_ active regions. The thicker the flake, the highest photoresponsivity is observed at lower illumination intensities, and its value corresponds very well with the response obtained for a light power of 100 μW before the WG. We are aware that only part of the light couples to the WG, and we assume that this coupling does not change with the illumination power. Therefore, the internal photoresponsivity of this type of detector is higher because it actually corresponds to lower lighting power, which is difficult to also estimate due to propagation loss in the WG. The calculations of propagation loss for varied distance between coupling light into WG and detector localization have been placed in Figure S1 in the Supporting Information.

A negative photoresponse, *i.e.*, lower current after illumination, is observed at higher laser powers, and we attribute this observation to sample degradation or other phenomena typical for van der Waals crystals. In general, we observed that thinner flakes degrade more quickly. To illustrate this phenomenon, Figure 6a includes the results for a photodetector with a thinner *n*-type flake exfoliated from the same MoS_2_ crystal. It is clearly visible that for a thinner flake, a negative *P*_R_ begins to appear even at lower laser powers. A larger *P*_R_ is attributed to the enhanced absorption of a thicker MoS_2_ flake and the stronger optical mode confinement in the MoS_2_ flake. This effect was noticeable for *n*-MoS_2_ as well as for *p*-MoS_2_. Systematic analysis of the effect of MoS_2_ thickness on *P*_R_ is complicated in this case because we observed that *P*_R_ also depends on flake size, which is difficult to control with mechanical exfoliation. Additionally, we observed that *P*_R_ of the detector depends on the position of the Pt contact pads. They cannot be located too far from the WG, *i.e.*, or too far from the unilluminated part of the flake.

**Figure 7:**
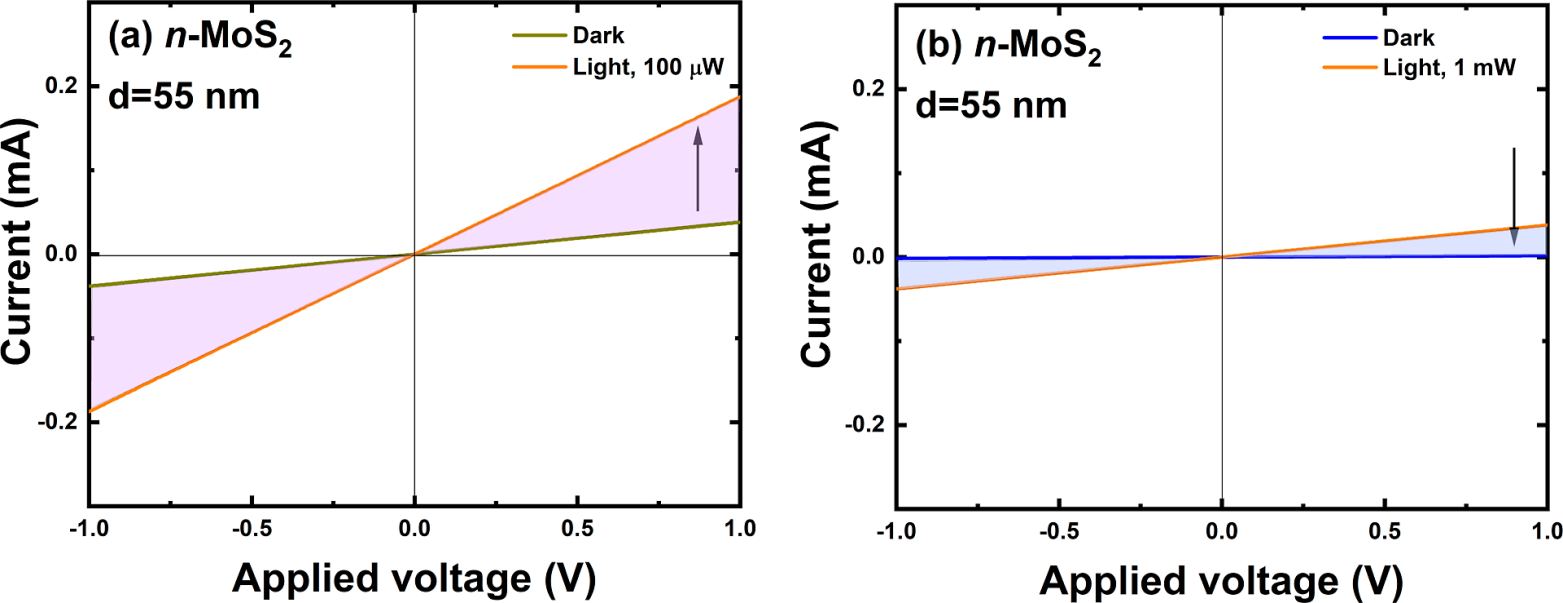
Optoelectrical photodetector characterization for MoS_2_/WG (flake thickness *d* = 55 nm): (a) light source power 100 μW and (b) light source power 1 mW.

To check the effect of flake degradation at higher WG excitation power, the *I*–*V* characteristics were measured for the WG excitation power of 100 μW and 1 mW, see Figure 7. The measured *I*–*V* characteristics at 1 mW in *I*_light_ condition clearly show a significant drop in current, which is related with flake degradation. However, it is worth emphasizing that the lighting powers at which we observed degradation of the active area of our detectors are very significant, and we will not expect such powers with these types of detectors. The light intensity regime in on-chip WG systems is at least 2 orders of magnitude lower, and in this regime, the proposed detector architecture gives a positive and very high response.

The performed measurements show that the value of photoresponsivity is most dependent on the flake thickness, where the thickness significantly affects the amount of current passing through the flake. We also noticed that the effect of changing conductivity after coupling light into the WG in thin and few-layer flakes is hardly noticeable.

**Figure 8:**
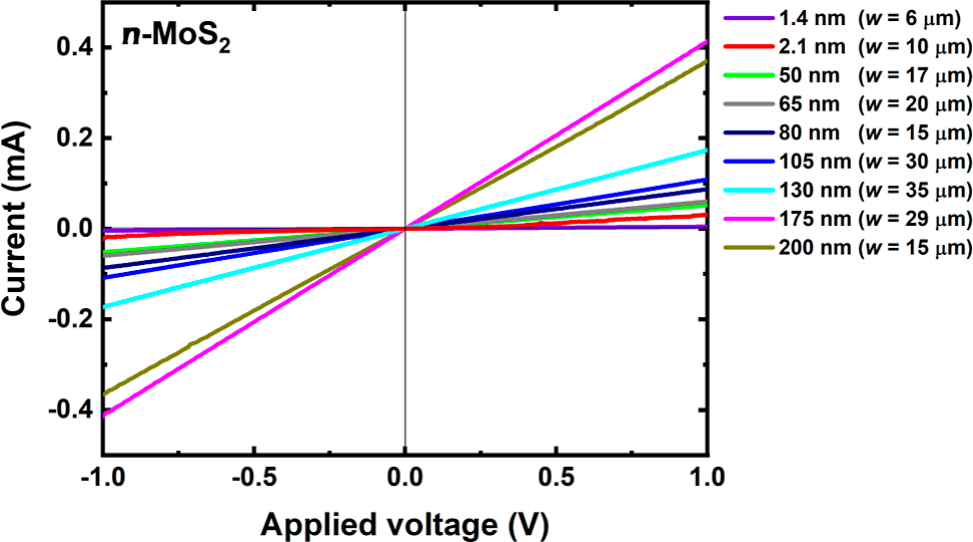
Thickness-dependent characteristics for the *n*-MoS_2_ devices under dark conditions. The flake width (*w*) in the fabricated devices was varied from 6 to 35 μm.

To show that the current flowing through the flake depends very much on its size, including thickness, *I*–*V* characteristics for various flakes were collected and are shown in Figure 8. All measurements were performed with and without WG illumination, but measurements with WG illumination are not shown in this figure because they cannot be easily compared since more than one flake was deposited on some WGs. The thicknesses (*d*) of the exfoliated flakes varied from 1.4 to 200 nm, and the flake width (*w*) was in the range of 6–35 μm. In this series of samples, only the samples with the smallest thickness are in the regime where thickness-related changes in the electronic band structure and optical properties of MoS_2_ can be expected, and hence, this aspect is neglected in this comparison. In the experiment, apart from changing the flake thickness, the other fabrication parameters were constant. From Figure 8, we can observe that the lowest conductivity (lower *I*_dark_) was measured for the thinnest flake, and the highest conductivity was obtained for the thicker flake as was expected in this case. Since the length and width of the flake are not preserved in this series of samples for obvious reasons resulting from the method of producing these types of samples, and they also affect the conductivity of the flake, the slopes of these characteristics are not completely systematic with respect to the flake thickness. Nevertheless, the effect of increasing current with an increasing flake thickness is clearly visible and is caused by greater light absorption in the MoS_2_ layer. From the point of view of the device, this is very important because larger currents are easier to measure.

For the fabricated MoS_2_ photodetector, the measurement to determine the conductivity was performed as described below. To obtain the photoresponse, the device was measured under the light–dark–light conditions with an interval period of 180 s; see Figure S2 in the Supporting Information. We observe that the current enhances upon coupling light into the WG and returns to the initial current level when the light is switched off. Therefore, when light is sucked from the WG into the flake, the photoinduced desorption of the O_2_ and H_2_O molecules from the flake surface does not appear. It is possible that the main reason is that the flake is excited by light from the WG and not directly from the top of the flake. However, we can see that this issue requires further research and experimental measurements, for instance, in pressure.

### 2.4 Spectroscopic Measurements

**Figure 9:**
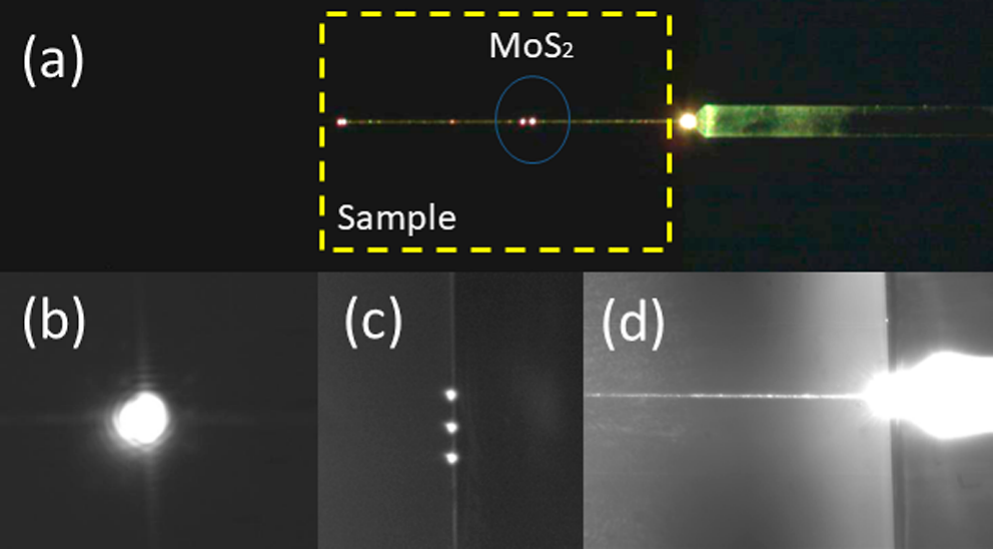
MoS_2_/sol–gel SiO_*x*_:TiO_*y*_ WG excitation. (a) Top view of the WG excitation on the sample. (b,c) Signal at the output of a WG (series of WGs). (d) View of the fiber tip during WG excitation.

**Figure 10:**
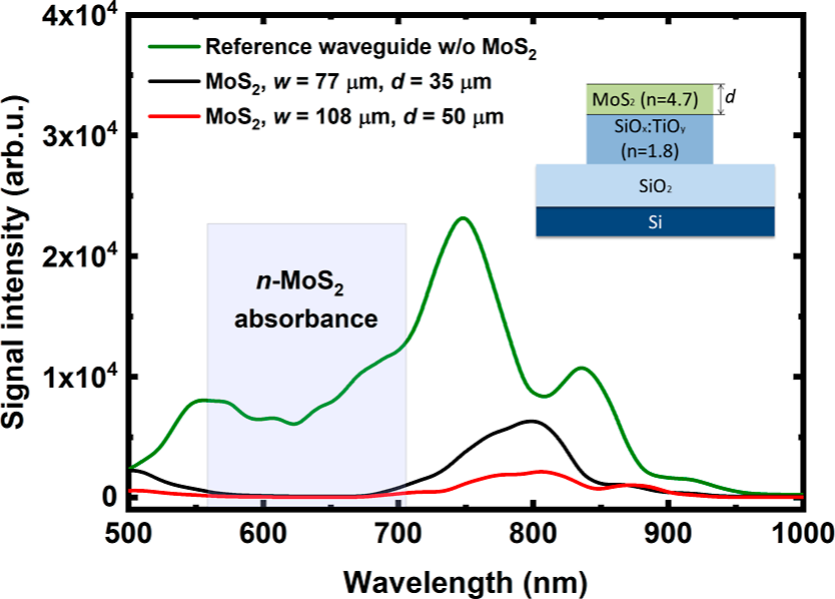
Transmission spectra of the sol–gel SiO_*x*_:TiO_*y*_ WG with/without MoS_2_. Transmission spectra were recorded for the reference WG (without flake on top of the WG), where the MoS_2_ flake thickness was *d* = 35 and 50 nm and flake width *w* = 77 and 108 μm.

To understand the absorption mechanism in the MoS_2_-based photodetector integrated with the sol–gel SiO_*x*_:TiO_*y*_ WG, we performed the spectroscopic characterization, where the transmission spectra were recorded for WGs with and without exfoliated MoS_2_ (see Figures 9 and 10).

After the transfer of the MoS_2_ flake to the WG, the transmission spectrum changed significantly. Here, we observed the effect of light being channeled out of the WG by the MoS_2_ flake due to its high refractive index (*n* = 4.774 at 0.586 μm for the bulk crystal) compared with the refractive index of the sol–gel WG, which is ∼1.8 at the same wavelength. Based on the spectroscopic measurements of our device, we can conclude that the light introduced into the WG is channeled out of the WG and almost completely absorbed by the flake, and hence, the manufactured device works as a photodetector. As the calculations presented later in this work show, the high contrast of the refractive index is responsible for the light escaping from the WG. Therefore, controlling the refractive index contrast between the WG and the active area of the detector is a natural way to control the amount of light absorbed by the active area and further propagating in the optical WG.

In our device, the WG (without a MoS_2_ flake) is multimode. The transmission loss for each mode is affected primarily by side-wall (TE-modes) and top-wall (TM modes) roughness. The channel (*i.e.*, the WG) width and height influence the number of guided modes and in such a way impact the overall transmission (Figure S3 in the Supporting Information). In our case, the transmission loss for the fundamental transverse electric (TE) mode estimated with a streak method was at the level below 1 dB/μm. To estimate these losses with better accuracy, another approach is advisable, and this may be extraction of the WG’s propagation loss using a cutback approach.^65−68^

### 2.5 Modification of the MoS_2_/Sol–Gel SiO_*x*_:TiO_*y*_ Photodetector by a Low-Refractive Index Separating Layer

**Figure 11:**
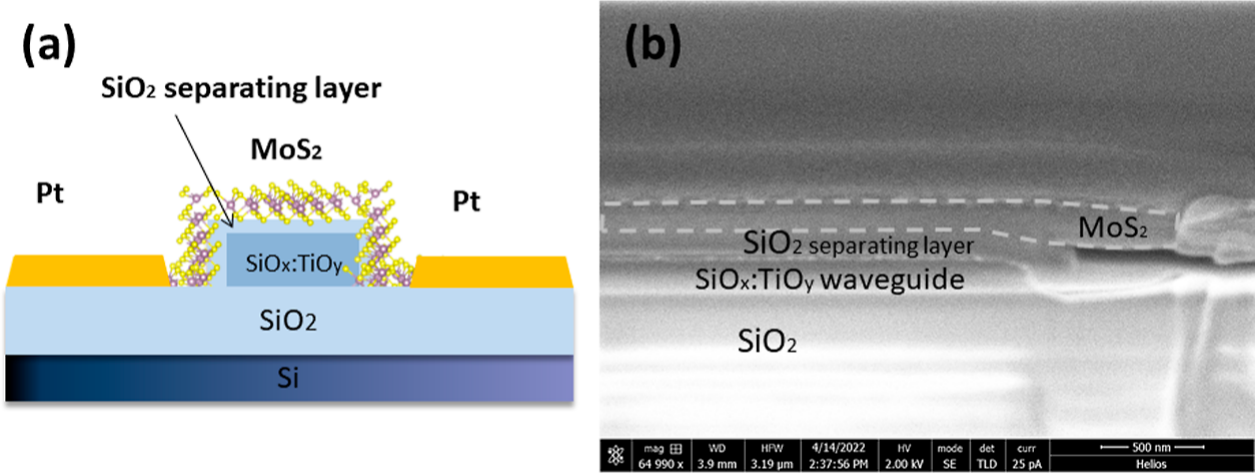
(a) Schematic cross-section of modified photodetector with a SiO_2_ separating layer. (b) SEM image of the cross-section of the photodetector: visible layers of MoS_2_, SiO_2_, and SiO_*x*_:TiO_*y*_ WG shape.

To control the amount of channeled out light from the WG, in the presence of MoS_2_ flake, a silicon dioxide (SiO_2_) layer was introduced between the WG and the active part made of MoS_2_, see Figure 11. The thicknesses of MoS_2_, SiO_2_ separating layer, and sol–gel SiO_*x*_:TiO_*y*_ WG were 264, 120, and 313 nm, respectively.

**Figure 12:**
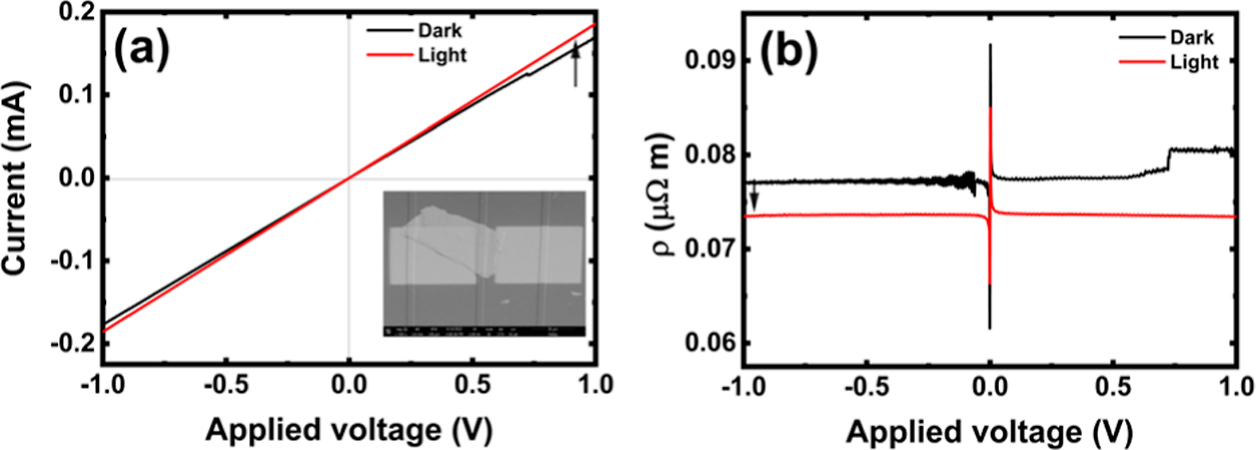
Optoelectrical photodetector characterization. (a) *I*–*V* characteristics for the *n*-MoS_2_/SiO_2_/WG (flake thickness *d* = 264 nm). Inset presents the SEM image of the modified photodetector with a SiO_2_ separating layer. (b) Calculated *ρ–V* characteristics for *n*-MoS_2_/SiO_2_/WG.

Figure 12 shows the optoelectrical characterization results for the photodetector with a SiO_2_ separating layer.

**Figure 13:**
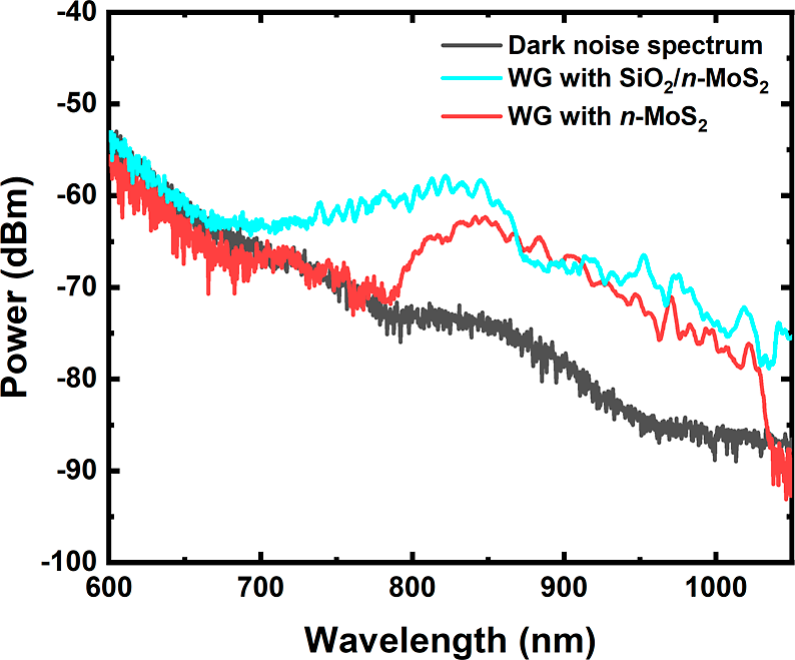
Output spectrum comparison of the reference WG (without *n*-MoS_2_ flake and SiO_2_ separation layer) and WGs with/without the *n*-MoS_2_ and SiO_2_ separating layer.

The *I*–*V* characteristic with/without the introduction of light into the WG proves that the modified device works as a photodetector, *i.e.*, light is channeled out from the WG, absorbed by the active part of the detector, and causes a current increase. Comparison of the photocurrent for a device with/without a SiO_2_ separating layer shows how this spacer weakens the light extraction from the WG. Still, for such a comparison, it is necessary to ensure the same geometric dimensions of the active part of the detector, and this is not easy in mechanical exfoliation. Therefore, for this purpose, measurements of light transmission spectra through the WG with/without MoS_2_ were performed and compared for the reference and modified devices, see Figure 13. Additionally, the transmission spectrum for the WG itself and the reference signal from the excitation source are shown in this figure.

Comparing these spectra, it is visible that the introduction of the SiO_2_ separating layer significantly modifies the transmission spectrum, *i.e.*, increasing the transmission of light through the WG compared to the device without this layer. This proves that the engineering of the refractive index at the WG/active-part junction via a SiO_2_ layer is a way to control the amount of light channeled out of the WG by MoS_2_.

### 2.6 Calculations of Propagation Losses in a MoS_2_-Based Sol–Gel WG Photodetector

**Figure 14:**
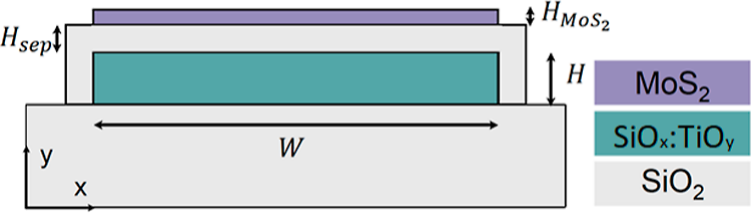
Cross-section of the SiO_*x*_:TiO_*y*_ WG with MoS_2_ and SiO_2_ separation layer (*H*—waveguide height; *W*—waveguide width; *H*_MoS_2__—MoS_2_ layer height; *H*_sep_—separation layer height; *n*_eff_—effective index; and *L*_s_—propagation loss).

According to the experimental results, a numerical model was developed for a MoS_2_ photodetector integrated with a sol–gel WG. Additionally, we estimated the expected propagation loss due to MoS_2_ and calculated these losses for the device with a SiO_2_ separating layer using finite element modeling in COMSOL Multiphysics. Figure 14 shows a schematic cross-section of the sol–gel SiO_*x*_:TiO_*y*_ WG with the MoS_2_ and SiO_2_ separating layer in COMSOL. Since the polarization of light is assumed in the simulations, we analyzed both TE- and (transverse magnetic) TM-polarized light. The experiment was carried out for unpolarized light, *i.e.*, light containing both TE and TM polarizations, and therefore, the analysis of these two polarizations is completely justified and is the best solution in such a case. This approach is also supported by the fact that light with a given polarization (TE or TM) may lose its polarization after passing through the WG and reaching the area with the MoS_2_ flake due to WG imperfections (for instance, the roughness of the side walls of the WG). This is an additional issue related to the SiO_*x*_:TiO_*y*_ WG that is not considered in this work. Therefore, the experiment was performed for unpolarized light.

The examples of the calculated modes (TE_0_, TE_8_, TM_0_, and TM_5_) for the SiO_*x*_:TiO_*y*_ WG with the MoS_2_ flake and device with a SiO_2_ layer (thickness 0, 120 and 240 nm) are presented in Figures S4−S7 in the Supporting Information. Figures S4−S7 show simulations for a wavelength of 684 nm to demonstrate the modal field distributions for different device arrangements. This wavelength was selected arbitrarily close to the long-wavelength limit of the absorbance range. Based on these simulations, it is clear that the thickness of the SiO_2_ layer is crucial in this type of device. MoS_2_ has the refractive index much higher than SiO_*x*_:TiO_*y*_ and therefore causes modal field dislocation toward the flake. In the simulation, we do not take into account scattering loss due to the side and top WG’s surface roughness, so the resultant calculated modal transmission loss is due to the confinement loss (which is negligibly small below the cutoff wavelength) and absorption of modal field overlapping with MoS_2_.

The separating layer prevents this overlap and is more efficient when the separation layer is thicker (for instance, 240 nm), as can be seen in Figure S7.

**Figure 15:**
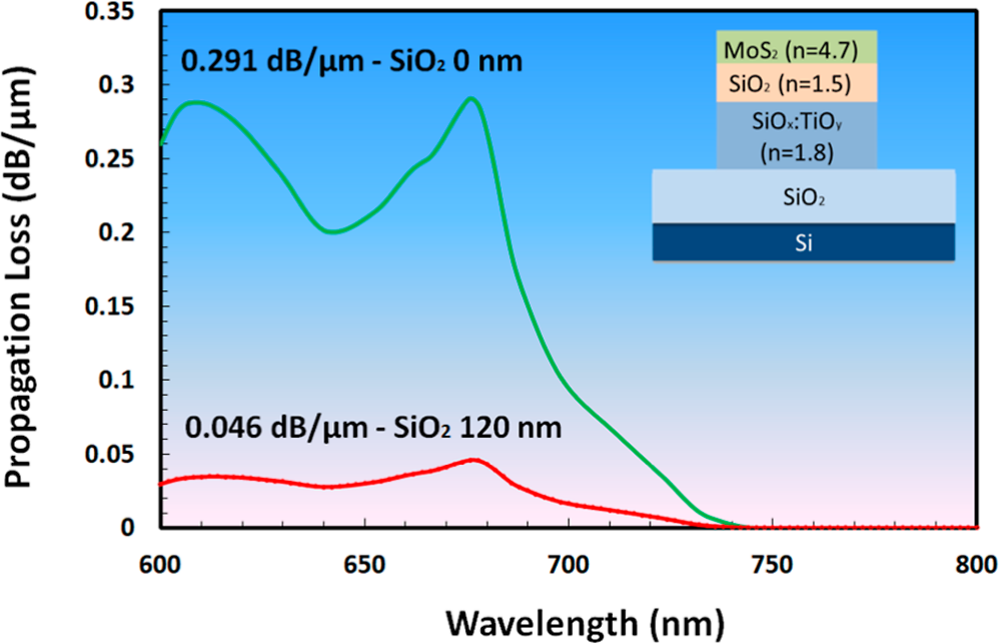
Calculated propagation losses for the WG (*W* = 5 μm and *H* = 0.3 μm) with/without the SiO_2_ separating layer (*H*_sep_ = 120 nm) as a function of wavelength. Losses were presented for the TE_0_ mode.

The propagation losses were calculated for two devices with and without the SiO_2_ separating layer (Figure 15). The calculated values of losses at 675 nm for the MoS_2_-based device with/without the SiO_2_ separating layer are 0.046 dB/μm (calculated value for SiO_2_ thickness 120 nm) and 0.291 dB/μm, respectively. Here, losses may be associated with flake absorption and the edge scattering effect due to MoS_2_.

The obtained loss values for the sol–gel WG with MoS_2_ are slightly lower than those for other material platforms such as SiN^59^ and Si.^19,62,63^ Additionally, the insertion of a SiO_2_ separating layer reduces the sol–gel WG’s propagation losses, thereby creating a photodetector on integrated circuits without introducing significant losses.^64^

## 3 Conclusions

In conclusion, we realized a photodetector with new device designs consisting of a sol–gel SiO_*x*_:TiO_*y*_ WG and mechanically exfoliated *n*- and *p*-type MoS_2_ flakes, enabling interactions between the strongly absorbing MoS_2_ flakes and tightly confined optical modes in the WG. When the WG is illuminated with a broad-band supercontinuum light spectrum with a power of 100 μW, the sensitivities of our devices, at the *V*_gs_ = 2 V, reach a maximum value of ∼3 × 10^−1^ and ∼4 × 10^−3^ A W^−1^ for *n*- and *p*-type MoS_2_ active regions, respectively. From the spectroscopic measurements of our fabricated devices, we observed that the light entering the WG is almost completely channeled out by the MoS_2_ flake because its refractive index is much higher than the refractive index of the WG (∼4 vs ∼1.8). The proposed detector design based on the high contrast of the refractive index between the WG and the active part can be used in another material system, and in this context, the presented results are of great importance even if this detector is not yet completely optimized and characterized. Additionally, it was shown that the amount of channeled out light could be controlled by the SiO_2_ separating layer between the WG and the MoS_2_ flake. These experimental observations are confirmed by simulations of light propagation in such devices. Both experimental studies and simulations clearly show that the proposed device can work as a sensitive photodetector. Additionally, such flakes on WGs, separated by a SiO_2_ layer, absorb only a smaller fraction of the propagating light, which can work as readers of information transmitted through the WG. Therefore, further work on this type of device is very interesting and promising for integrated photonic circuits.

## 4 Experimental Section

### 4.1 Sample Preparation

#### 4.1.1 Synthesis of SiO_*x*_:TiO_*y*_ Sol–Gel

Acid-catalyzed silica–titania SiO_*x*_:TiO_*y*_ sol was prepared from tetraethyl orthosilicate (TEOS, Sigma-Aldrich) and titanium(IV) ethoxide (TET, Sigma-Aldrich). TEOS and TET were used as precursors of silica and titania, respectively. Hydrochloric acid (HCl 36%, Avantor) was used as the catalyst, and anhydrous ethanol (EtOH 99.8%, Avantor) was used as the solvent. The molar ratio of TEOS to TET was 1:1. The procedure of synthesis of the silica–titania sol is described in ref (57). After synthesis, sol–gel layers were prepared via the dip-coating technique, followed by annealing in an oven at 500 °C for 1 h. The thickness of the obtained sol–gel layers was controlled during deposition by changing the withdrawal speed of the substrate.

#### 4.1.2 Device Fabrication

First, the device was fabricated on a commercial silicon dioxide-on-silicon wafer covered on top by 3000 nm-thick SiO_2_ (MicroChemicals GmbH) and sol–gel SiO_*x*_:TiO_*y*_ layers with a thickness of 300 nm. The planar WGs were patterned using photolithography with a positive photoresist (ma-P 1215; Microresist Technology). The patterned features were further etched using an inductively coupled plasma etching tool. The photoresist mask was removed using an acetone wash and short oxygen plasma cleaning. Each sample was cleaned with acetone and isopropanol and dried using nitrogen gas.

Thin flakes of van der Waals crystals were obtained via mechanical exfoliation by using the Scotch tape technique. Thin MoS_2_ flakes were obtained from bulk single crystals (2D Semiconductors Co., Ltd.) via mechanical exfoliation (Schubert Technologies tape, Gel-Pak PDMS). Both *n*-type and *p*-type materials were used to produce the detectors. The *n*-type material was nominally undoped MoS_2_, which due to S vacancies has *n*-type conductivity.^69^ The *p*-type material was niobium-doped MoS_2_. In both materials, carrier concentrations were several times 10^17^ cm^−3^. The MoS_2_ flakes on the samples were verified by the color contrast between MoS_2_ and SiO_2_/Si using an optical microscope (Delta Optical MET-200-RF/TRF). The few-layered MoS_2_ flakes were transferred to the top of the SiO_*x*_:TiO_*y*_ WGs by using a micromanipulator equipped with an optical microscope. Next, 50 nm-thick platinum metal pads were prepared in a FEI Helios HanoLab microscope using GIS deposition and FIB source.

### 4.2 Microscopic Analysis and Thickness Measurements

SEM images and FIB cross-section were obtained using a Helios NanoLab 660 microscope equipped with the Schottky electron source, Ga^+^ ion source, and GIS for platinum deposition. The FIB cross-section was made by ion beam milling. All optical microscopy images were obtained using a Leica DM300. All electrical and spectroscopic measurements were performed under ambient air and laboratory conditions.

The thickness measurements of exfoliated flakes were performed with a Bruker surface profilometer with 100 nm stylus and a vertical resolution of 1 nm for 65.5 μm scan range, given in the specifications of this equipment. According to our previous research and experience in using this profilometer for the exfoliated van der Waals crystals, this allows us to estimate the flake thickness with an accuracy of single nanometers, which is sufficient in this case. The flake length (*L*) was estimated based on observations using a Lecia DM300 optical microscope.

### 4.3 Electrical and Photoresponse Measurements

Electrical measurements of the photodetector were performed by using a Keysight B2901A precision source meter. The photoresponse of the device was measured using a broad-band tungsten Light Source (Thorlabs SLS201L) with an SMA fiber. The wavelength range of 400−2200 nm and a source power of 100 μW were applied for all measurements.

### 4.4 Spectroscopic Characterization of the Sol–Gel WGs

**Figure 16:**
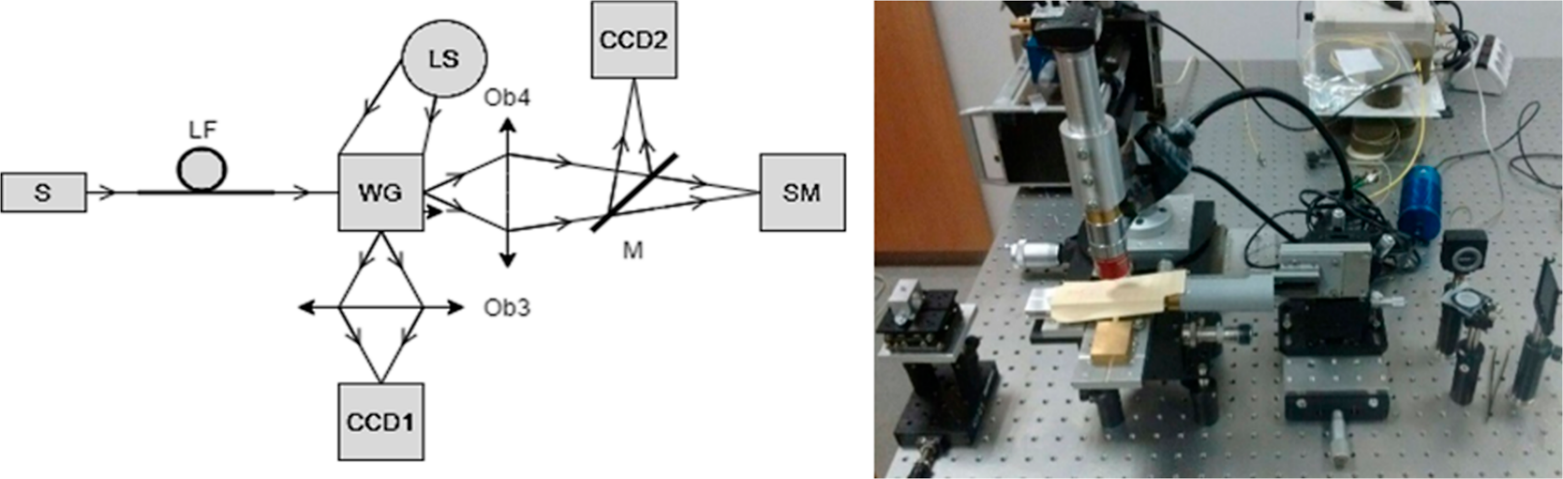
Schematic of the experimental setup (S—light source, LF—lensed fiber, WG—waveguide sample, LS—overhead white light source, Ob3, Ob4—objectives, CCD1, CCD2—cameras, M—mirror, and SM—spectrometer).

We used a measurement setup that made it possible to have a visual inspection of the investigated WG quality and quantitative assessment of WG excitation. A schematic of the setup component arrangement is shown in Figure 16. Initially, a visible light laser diode (S) was used to simplify the setup adjustment and testing. Analyzed WG samples exhibited small cross-sectional dimensions with a height in the range of 0.3 μm and a width of 5, 2, and 1.2 μm. To provide a sufficiently small spot size for adequate light coupling and precise excitation of individual WGs on the sample (Figure 16), a lensed fiber (LF) was used (a spot diameter of approximately 2.5 μm). A fiber optic illuminator (LS), camera CCD1, and objective Ob3 were employed for top-view observation of the WG samples. Camera CCD2 and Ob4 were used for WG output inspection. A mirror (M) was placed in the setup to enable the alternate acquisition of the light signal via a camera CCD2 or spectrometer (SM).

### 4.5 Calculations

Calculations were performed using COMSOL Multiphysics 6.1 software. The built-in Wave Optics module was employed to perform frequency-domain mode analysis following the general methodology described, for instance, in ref (70). We assumed the translational invariance of the examined WGs, limiting the Cartesian computational window to a 2D PML-truncated WG cross-section. We assumed that the leaky hybrid modes propagate in an out-of-plane direction. Analyzed WGs are bounded from the top by the air with a refractive index set to 1. We considered material dispersions for SiO_2_, SiO_*x*_:TiO_*y*_, and MoS_2_. The modes were computed by using the ARPACK mode solver. Power loss expressed in dB/μm was obtained based on the imaginary part of complex propagation constants.

Searching for the complex propagation constants and modal field distributions in the PML-truncated model involving lossy materials results in the nonlinear boundary eigenvalue problem to be solved. To perform this task effectively, we used the simplified model to calculate guess values for the propagation constants accurately. A computational window in the simplified model was truncated with the scattering boundary conditions and had a significantly smaller area. Simplified model refractive indices of the TE_0_ mode were used to “search for modes around” in the full model. Wavelengths within the 600−800 nm range with a 2 nm step were analyzed. For each wavelength, the propagation constants of the materials were computed, and a mode analysis was performed with and without the MoS_2_ layer. The mesh size was set to 0.175 nm () in the MoS_2_ layer and approximately 15 nm in the core of the WG. We considered material dispersions for SiO_2_, SiO_*x*_:TiO_*y*_, and MoS_2_.

## Supporting Information Available

Calculations of propagation loss for varied distance between light coupling into WG and detector localization; measured photocurrent in the light–dark–light state under ambient conditions; output spectra comparison for WGs with varied channel width 1.2–20 μm; example modes for SiO_*x*_:TiO_*y*_ WG with the MoS_2_ monolayer and 50 nm flake; and example modes for SiO_*x*_:TiO_*y*_ WG with MoS_2_ flake and SiO_2_ layer (0, 120, and 240 nm) (PDF)
