# Supplementary material for: Waveguide-Coupled Light Photodetector Based on Two-Dimensional Molybdenum Disulfide
Source: ACS Appl Mater Interfaces. 2024 May 25;16(22):28874–85. doi: 10.1021/acsami.4c04854 (PMC11163399; doi:10.1021/acsami.4c04854)
Supplement: Supplementary file 1 — am4c04854_si_001.pdf [file am4c04854_si_001.pdf]

Supporting Information for

Waveguide-coupled Light Photodetector Based on Two-Dimensional Molybdenum Disulfide

Daria Hlushchenko[1,2*], *Jacek Olszewski*[2], *Tadeusz Martynkien*[2], *Michał Łukomski*[2], *Karolina Gemza*[2], *Pawel Karasiński*[3], *Magdalena Zięba*[3], *Tomasz Baraniecki*[1], *Łukasz Duda*[1,4], *Alicja Bachmatiuk*[1], *Małgorzata Guzik*[1,4], *Robert Kudrawiec*[1,2*]

[1]Lukasiewicz Research Network-PORT Polish Center for Technology Development, ul. Stablowicka 147, 54-066, Wroclaw, Poland

[2]Wroclaw University of Science and Technology, Faculty of Fundamental Problems of Science and Technology, Wybrzeze Wyspianskiego 27, 50-370 Wroclaw, Poland

[3]Silesian University of Technology, Department of Optoelectronics, ul. B. Krzywoustego 2, 44-100, Gliwice, Poland

[4]University of Wroclaw, Faculty of Chemistry, ul. F. Joliot-Curie 14, 50-383 Wroclaw, Poland

Corresponding authors: daria.hlushchenko@pwr.edu.pl, robert.kudrawiec@pwr.edu.pl

The calculations of propagation loss for varied distance between light coupling into waveguide and detector localization are shown in the Figure S7. The propagation loss was calculated with streak method.[67] According to this method, the intensity of the scattered light at each point in the streak is proportional to the intensity of the light propagating at that point in the waveguide layer. The obtained propagation loss value is 0.52 dB/cm (for distance 0.35 cm); 2.26 dB/cm (for distance 0.4 cm); 4.75 dB/com for distance 0.5 cm.

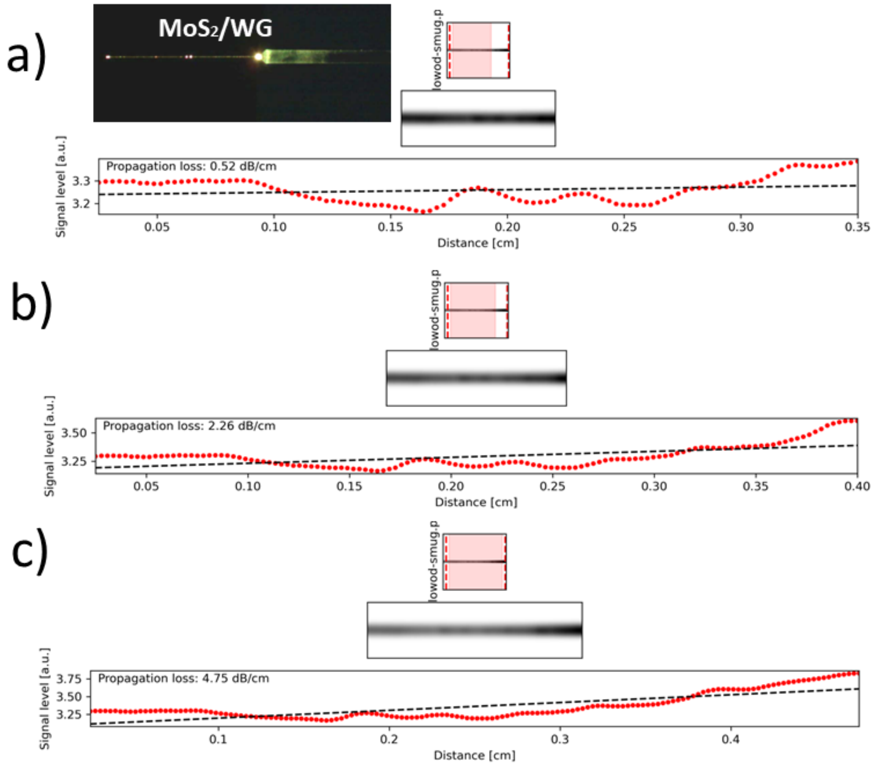

Figure S1. Calculated propagation loss for MoS_2/sol-gel waveguide for distance between coupling light into waveguide and detector localization: a) 0.35 cm; b) 0.4 cm; c) 0.5 cm.

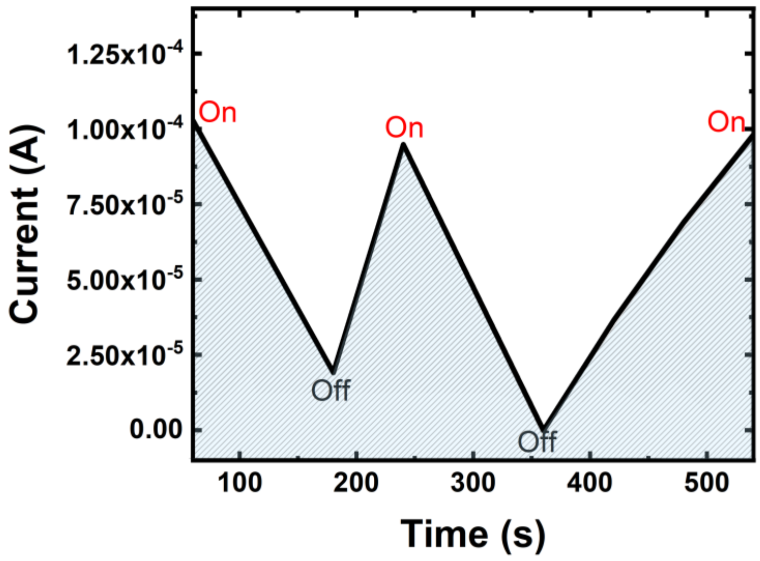

Figure S2. Measured photocurrent in the light-dark-light state under ambient conditions.

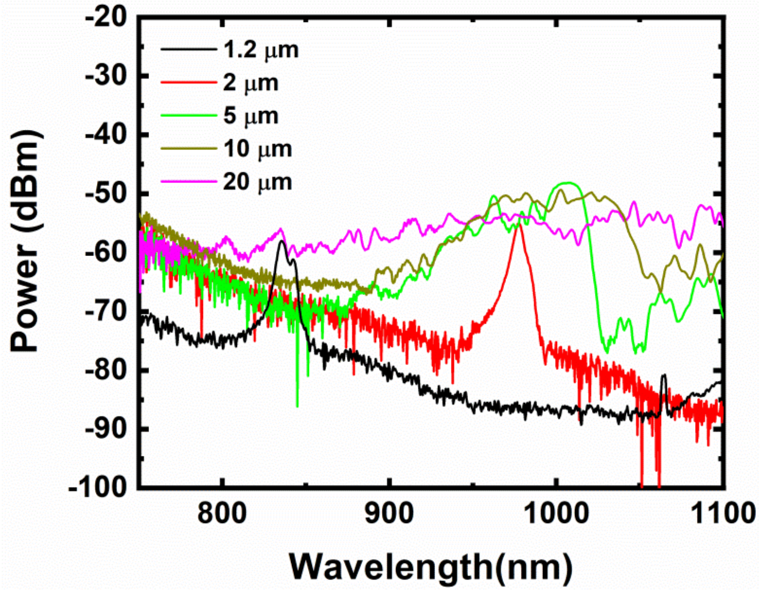

Figure S3. Output spectra comparison for waveguides with varied channel width. The presented spectra for waveguides with width 1.2-20 μm.

The distribution of electric field norm for the fundamental TE/TM modes and selected higher order mode in the TiO_x:SiO_y waveguide, waveguide with MoS_2 flake on top, and waveguide with SiO_2 layer separating MoS_2 flake from the waveguide were presented in Figures S4-S7 (ESM).

MoS_2 has the refractive index much higher than the TiO_x:SiO_y and therefore causes modal field dislocation toward the flake. In the simulation we do not take into account scattering loss due to the side and top waveguide's surface roughness so the resultant calculated modal transmission loss is due to the confinement loss (which is negligibly small below the cut-off wavelength) and absorption of modal field overlapping with the MoS_2. Separating layer prevent this overlap and is more efficient when the separation layer is thicker (for instance 240 nm), as can be seen on the Figure S7.

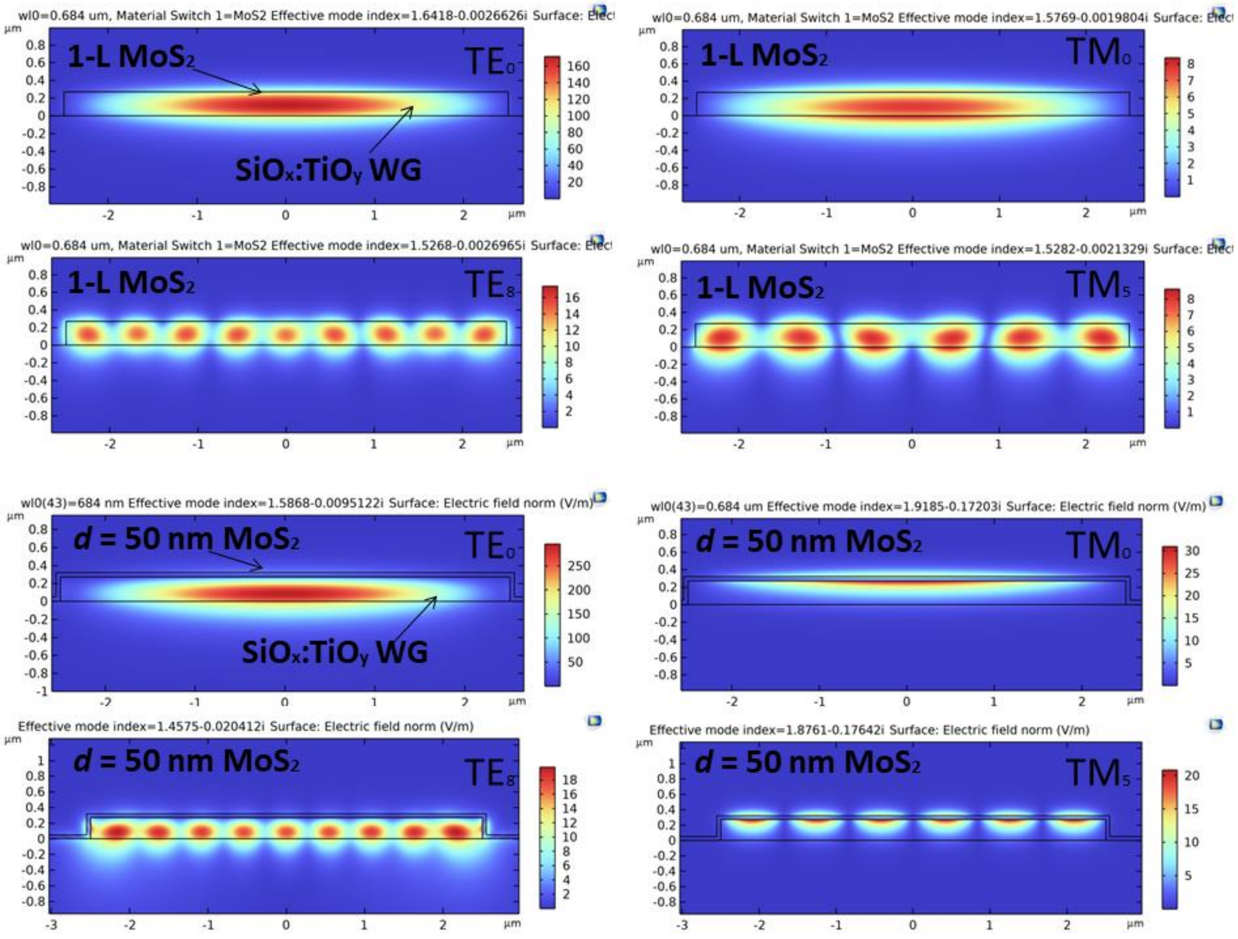

Figure S4. Example modes for SiO_x:TiO_y waveguide with MoS_2 flake. Modes are presented for MoS_2 monolayer (TE_0, TM_0, TE_8, TM_5) and thick flake with thickness of 50 nm (TE_0, TM_0) at a arbitrarily selected wavelength of 684 nm.

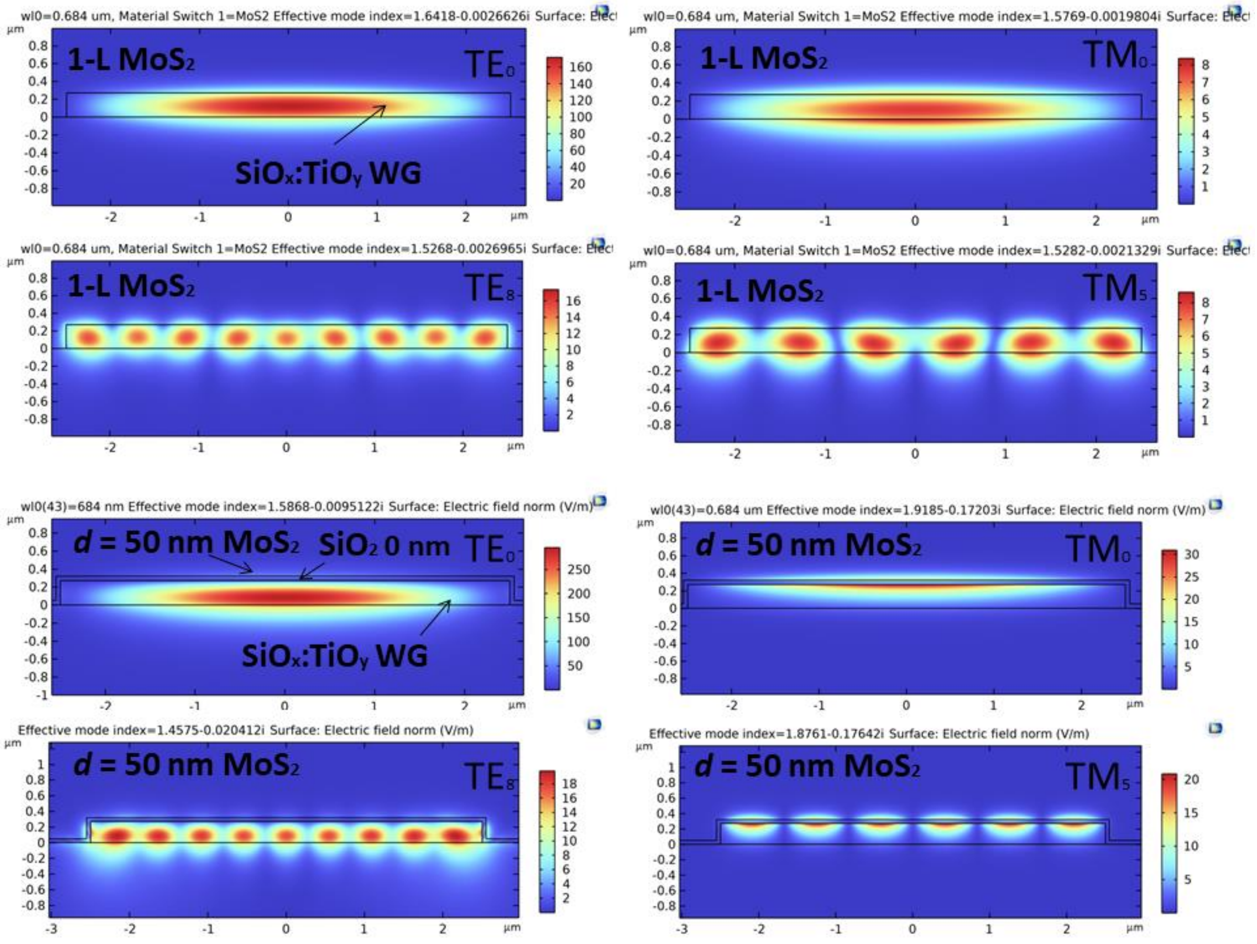

Figure S5. Example modes for SiO_x:TiO_y waveguide with MoS_2 flake and SiO_2 layer (0 nm). Modes are presented for MoS_2 monolayer (TE_0, TM_0, TE_8, TM_5) and thick flake with thickness of 50 nm (TE_0, TM_0) at a arbitrarily selected wavelength of 684 nm.

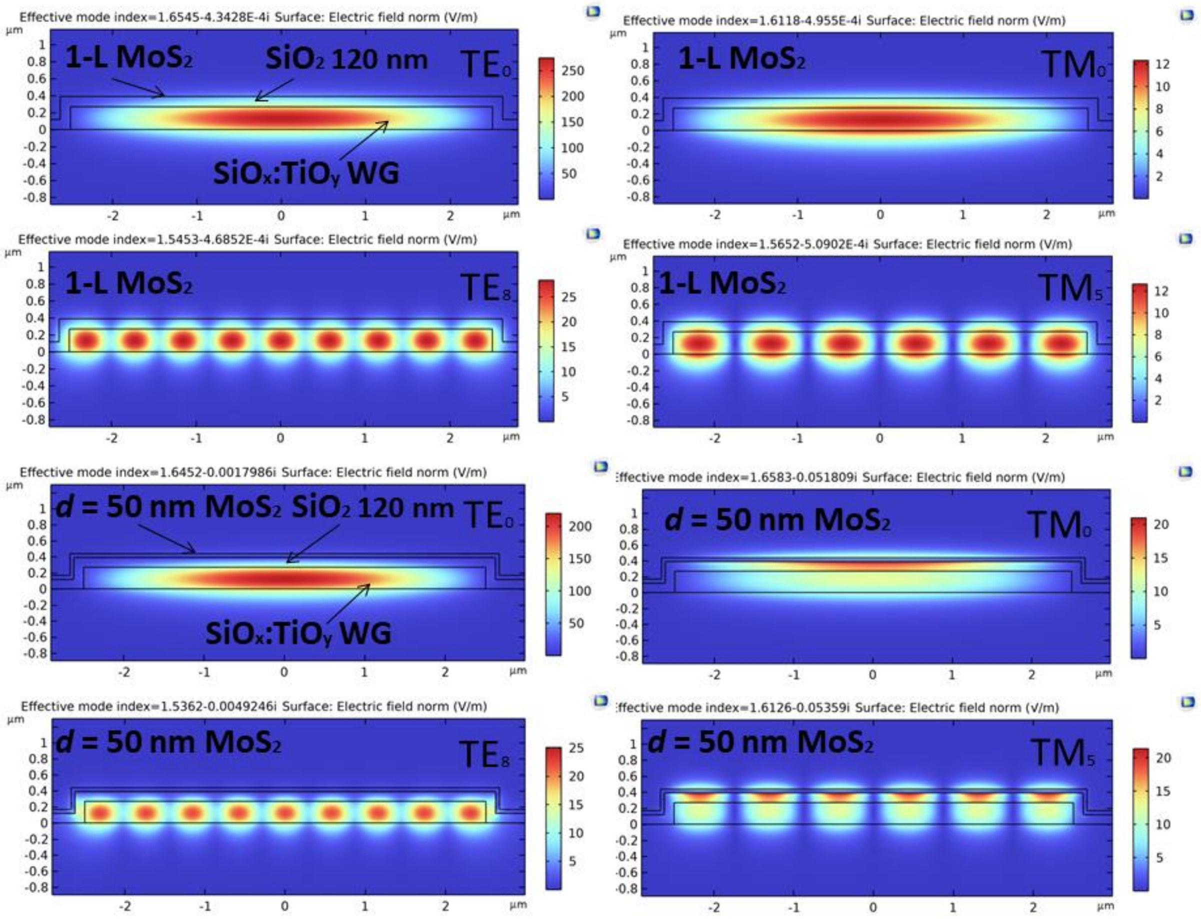

Figure S6. Example modes for SiO_x:TiO_y waveguide with MoS_2 flake and SiO_2 layer (120 nm). Modes are presented for MoS_2 monolayer (TE_0, TM_0, TE_8, TM_5) and thick flake with thickness of 50 nm (TE_0, TM_0) at a arbitrarily selected wavelength of 684 nm.

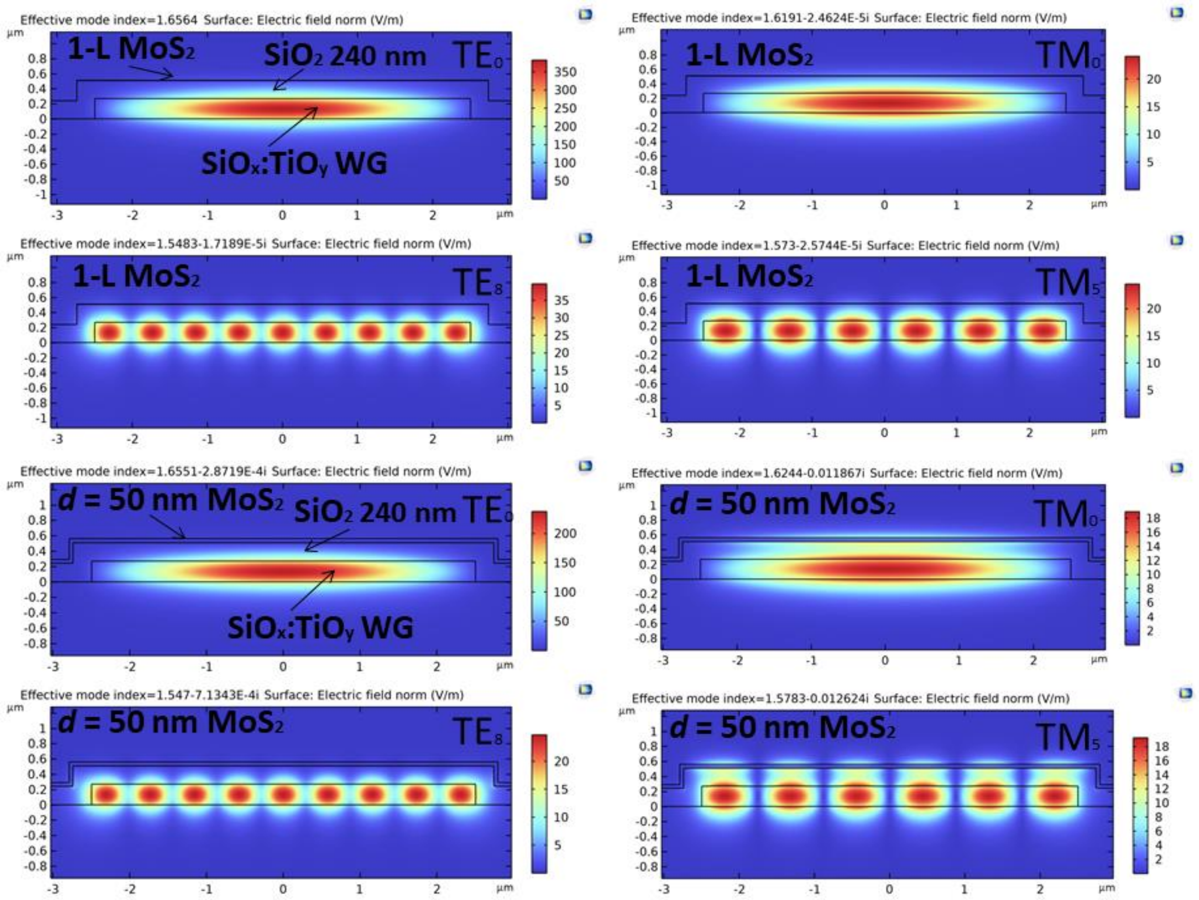

Figure S7. Example modes for SiO_x:TiO_y waveguide with MoS_2 flake and SiO_2 layer (240 nm). Modes are presented for MoS_2 monolayer (TE_0, TM_0, TE_8, TM_5) and thick flake with thickness of 50 nm (TE_0, TM_0) at a arbitrarily selected wavelength of 684 nm.
